# Bis(2-{2-[2-(benzyl­carbamo­yl)phen­oxy]acetamido}­eth­yl)ammonium nitrate ethanol disolvate

**DOI:** 10.1107/S1600536810052670

**Published:** 2010-12-24

**Authors:** Jiaji Liu, Xiaoliang Tang, Zhengdan Lu, Guolin Zhang, Weisheng Liu

**Affiliations:** a Key Laboratory of Nonferrous Metal Chemistry and Resources Utilization of Gansu Province, College of Chemistry and Chemical Engineering and State Key Laboratory of Applied Organic Chemistry, Lanzhou University, Lanzhou 730000, People’s Republic of China

## Abstract

In the title compound, C_36_H_40_N_5_O_6_^+^·NO_3_^−^·2C_2_H_5_OH, the nitrate anion is disordered over the two orientations of equal occupancy while the solvent mol­ecule reveals large displacement parameters. The cation is formed by protonation of the N atom of a secondary amine in the middle of the flexible chain and the whole compound has crystallographically imposed *C*-2 symmetry with the crystallographic *b* axis. An O atom of the nitrate anion links the acidic H atoms of the cation *via* N—H⋯O hydrogen bonding. In addition, neighbouring cations are connected by inter­molecular N—H⋯O hydrogen bonds and π–π inter­actions between the benzamide groups of the cations [centroid–centroid distance = 4.000 (3) Å], forming a chain along [001]. The ethanol solvent mol­ecules are arranged on the side of the chain through O—H⋯O hydrogen bonds.

## Related literature

Luminescent lanthanide complexes have attracted intense research inter­est due to their very narrow emission bands and large Stokes shifts, see: Wang *et al.* (2009 ▶); Bunzli & Piguet (2005 ▶); Stein & Wurzberg (1975 ▶). For amide-type open-chain ligands, see: Liu *et al.* (2009 ▶); Yi *et al.* (2007 ▶); Hamann *et al.* (2004 ▶). 
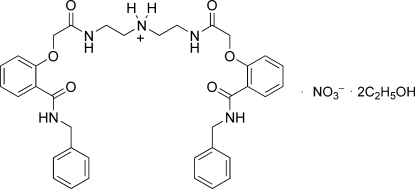


## Experimental

### 

#### Crystal data

C_36_H_40_N_5_O_6_^+^·NO_3_^−^·2C_2_H_6_O
*M* *_r_* = 792.88
Monoclinic, 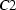

*a* = 16.978 (3) Å
*b* = 11.405 (2) Å
*c* = 11.164 (2) Å
β = 108.04 (3)°
*V* = 2055.6 (7) Å^3^
*Z* = 2
Mo *K*α radiation
μ = 0.09 mm^−1^
*T* = 296 K
0.22 × 0.18 × 0.17 mm

#### Data collection

Bruker SMART CCD area-detector diffractometer
Absorption correction: multi-scan (*SADABS*; Bruker, 1997 ▶) *T*_min_ = 0.980, *T*_max_ = 0.984
4901 measured reflections
1995 independent reflections
1334 reflections with *I* > 2σ(*I*)
*R*_int_ = 0.035

#### Refinement

*R*[*F* ^2^ > 2σ(*F* ^2^)] = 0.063
*wR*(*F* ^2^) = 0.190
*S* = 1.03
1995 reflections
277 parameters
45 restraints
H atoms treated by a mixture of independent and constrained refinement
Δρ_max_ = 0.26 e Å^−3^
Δρ_min_ = −0.32 e Å^−3^

### 

Data collection: *SMART* (Bruker, 1997 ▶); cell refinement: *SAINT* (Bruker, 1997 ▶); data reduction: *SAINT*; program(s) used to solve structure: *SHELXS97* (Sheldrick, 2008 ▶); program(s) used to refine structure: *SHELXL97* (Sheldrick, 2008 ▶); molecular graphics: *SHELXTL* (Sheldrick, 2008 ▶); software used to prepare material for publication: *SHELXTL*.

## Supplementary Material

Crystal structure: contains datablocks I, global. DOI: 10.1107/S1600536810052670/kp2291sup1.cif

Structure factors: contains datablocks I. DOI: 10.1107/S1600536810052670/kp2291Isup2.hkl

Additional supplementary materials:  crystallographic information; 3D view; checkCIF report

## Figures and Tables

**Table 1:** Hydrogen-bond geometry (Å, °)

*D*—H⋯*A*	*D*—H	H⋯*A*	*D*⋯*A*	*D*—H⋯*A*
N3—H*N*3⋯O5^i^	0.95 (4)	2.49 (5)	2.905 (9)	106 (3)
N3—H*N*3⋯O5^ii^	0.95 (4)	2.41 (5)	2.905 (9)	112 (3)
N3—H*N*3⋯O1^ii^	0.95 (4)	2.01 (4)	2.780 (3)	137 (4)
O4—H4*A*⋯O3^iii^	0.85	1.89	2.740 (11)	178
N1—H1*A*⋯O2	0.86	1.99	2.645 (5)	132
N2—H2*A*⋯O5^ii^	0.86	2.14	2.813 (9)	135
N2—H2*A*⋯O6^i^	0.86	2.35	3.185 (9)	163

Symmetry codes: (i) 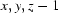; (ii) 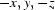; (iii) 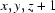.

**Fig. 1.**
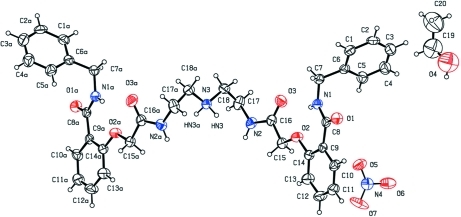
Molecular structure of the title compound. Displacement ellipsoids are drawn at the 30% probability level. Symmetry code used to generate the complete cation: -x, y, -1 - z.

**Fig. 2.**
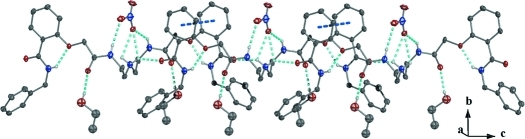
The hydrogen bonded chain dominates the crystal packing. Hydrogen bonds are shown as turquiose dashed lines. H atoms not involved in hydrogen bonding have been omitted for clarity. The π···π interactions between benzamide groups are shown as light-blue dashed lines.

## supplementary crystallographic information

### Comment

Luminescent lanthanide complexes have attracted intense research interest from both material and biological science mainly due to their very narrow emission bands and large Stokes shifts, etc (Wang *et al.*, 2009; Bunzli & Piguet, 2005; Stein & Wurzberg, 1975). Since the lanthanides have low absorptivities and poor quantum yields, fluorescence enhancement has generally been achieved through ligand sensitisation. Among various kinds of ligands, amide type open-chain ligands have drawn much attention in past ten years, mainly due to their flexible structure, selective coordinating capacity and hard binding sites, which could stabilise their lanthanide complexes, acquire novel coordination structure and shield the encapsulated ion from interaction with the surroundings (Liu *et al.*, 2009; Yi *et al.*, 2007; Hamann *et al.*, 2004). There is a need to prepare a new series of amide type ligands to further widen the scope of research on the chemical and physical properties of lanthanide complexes. In this paper, we present the structure of the title compound, a nitrate salt of a free ligand. The asymmetric unit comprises a half of cation C_36_H_40_N_5_O_6_^+^ nitrate anion, and ethanol molecule(Fig. 1). In C_36_H_40_N_5_O_6_^+^cation, the N atom of secondary amine in the middle of flexible chain is protonated adding more hydrogen bondind donor sites. An O atom of nitrate anion links the acidic H atoms and adjacent one of amide groups via N–H···O hydrogen bonding (Table 1) forming the S-shape organic cation. The carbonyl groups of benzamide at both ends of organic cation  are involved in intermolecular N–H···O hydrogen bonds connecting acidic H atoms of neighbouring C_36_H_40_N_5_O_6_^+^cations to form a chain. The chain is further stabilised by π–π interactions between the benzamide groups from cations (Fig. 2). Ethanol molecules are arranged on the side of the chain and linked the O atoms of the acetamide groups via O–H···O hydrogen bonds.

### Experimental

Ethyl 2-(2-(benzylcarbamoyl)phenoxy)acetate was firstly synthesised as a white solid *via* a simple prepared by reaction of *N*-benzylsalicylamide (2.27 g, 10.0 mmol) with ethyl chloroacetate (1.83 g, 15.0 mmol) in 80% yield using an excess amount of anhydrous potassium carbonate in refluxing acetone. Then the ligand, *N*,*N'*-iminodiethylenebis{[(2*'*-benzylaminoformyl)phenoxyl]acetamide}, was obtained in 18% yield by treating the ethyl 2-(2-(benzylcarbamoyl)phenoxy)acetate (1.88 g, 6 mmol) with 0.5 equiv of diethylenetriamine (0.258 g,2.5 mmol) in methanol at 333-338 K for 8 h (m.p. 372-374 K). Single crystals of the title compound suitable for X-ray analysis were obtained by slow evaporation of an ethanol solution (6 mL) containing Tb(NO_3_)_3_.6H_2_O (67.9 mg, 0.15 mmol) and *N*,*N'*-iminodiethylenebis{[(2*'*-benzylaminoformyl)phenoxyl]acetamide} (95.6 mg, 0.15 mmol) after two weeks at room temperature in 75% yield. The preparation was aimed to obtain an Tb(III) nitrate complex with the title ligand but instead the ligand was crystallised.

### Refinement

The nitrate group is disordered over two orientations where oxygen atoms of these two orientations revealed occupancies of 0.50 respectively. Furthermore, the anisotropic displacement parameters of the minor occupancy atoms C19, C20 and O4 of ethanol solvent were constrained by using the *SHELX* command ISOR and SIMU. The ammonium H atoms were located from the difference Fourier map and refined freely. H atoms were positioned geometrically with N—H = 0.86 for amide. All other H atoms were positioned geometrically with C—H = 0.93 and 0.97 Å for aromatic and methylene H atoms, respectively. They were constrained to ride on their parent atoms with *U*iso(H)= 1.2*U*eq(C) or 1.5 *U*eq(C).

### Crystal data

**Table:**

C_36_H_40_N_5_O_6_^+^·NO_3_^−^·2C_2_H_6_O	*F*(000) = 844
*M**_r_* = 792.88	*D*_x_ = 1.281 Mg m^−^^3^
Monoclinic, *C*2	Mo *K*α radiation, λ = 0.71073 Å
Hall symbol: C2y	Cell parameters from 1019 reflections
*a* = 16.978 (3) Å	θ = 2.5–20.9°
*b* = 11.405 (2) Å	µ = 0.09 mm^−^^1^
*c* = 11.164 (2) Å	*T* = 296 K
β = 108.04 (3)°	Block, colourless
*V* = 2055.6 (7) Å^3^	0.22 × 0.18 × 0.17 mm
*Z* = 2	

### Data collection

**Table:**

Bruker SMART CCD area-detector diffractometer	1995 independent reflections
Radiation source: fine-focus sealed tube	1334 reflections with *I* > 2σ(*I*)
graphite	*R*_int_ = 0.035
φ and ω scans	θ_max_ = 25.5°, θ_min_ = 2.2°
Absorption correction: multi-scan (*SADABS*; Bruker, 1997)	*h* = −20→20
*T*_min_ = 0.980, *T*_max_ = 0.984	*k* = −13→13
4901 measured reflections	*l* = −8→13

### Refinement

**Table:**

Refinement on *F*^2^	Primary atom site location: structure-invariant direct methods
Least-squares matrix: full	Secondary atom site location: difference Fourier map
*R*[*F*^2^ > 2σ(*F*^2^)] = 0.063	Hydrogen site location: inferred from neighbouring sites
*wR*(*F*^2^) = 0.190	H atoms treated by a mixture of independent and constrained refinement
*S* = 1.03	*w* = 1/[σ^2^(*F*_o_^2^) + (0.1204*P*)^2^] where *P* = (*F*_o_^2^ + 2*F*_c_^2^)/3
1995 reflections	(Δ/σ)_max_ < 0.001
277 parameters	Δρ_max_ = 0.26 e Å^−^^3^
45 restraints	Δρ_min_ = −0.32 e Å^−^^3^

### Special details

**Table:**

Geometry. All e.s.d.'s (except the e.s.d. in the dihedral angle between two l.s. planes) are estimated using the full covariance matrix. The cell e.s.d.'s are taken into account individually in the estimation of e.s.d.'s in distances, angles and torsion angles; correlations between e.s.d.'s in cell parameters are only used when they are defined by crystal symmetry. An approximate (isotropic) treatment of cell e.s.d.'s is used for estimating e.s.d.'s involving l.s. planes.
Refinement. Refinement of *F*^2^ against ALL reflections. The weighted *R*-factor *wR* and goodness of fit *S* are based on *F*^2^, conventional *R*-factors *R* are based on *F*, with *F* set to zero for negative *F*^2^. The threshold expression of *F*^2^ > σ(*F*^2^) is used only for calculating *R*-factors(gt) *etc*. and is not relevant to the choice of reflections for refinement. *R*-factors based on *F*^2^ are statistically about twice as large as those based on *F*, and *R*- factors based on ALL data will be even larger. The structure is non-centrosymmetric with only atoms lighter than silicon, MoKa measured Friedel data can not be used to determine absolute structure.

### Fractional atomic coordinates and isotropic or equivalent isotropic displacement parameters (Å^2^)

**Table:**

	*x*	*y*	*z*	*U*_iso_*/*U*_eq_	Occ. (<1)
C1	0.1027 (3)	0.7751 (5)	0.3251 (4)	0.0729 (14)	
H1	0.0513	0.8071	0.2823	0.088*	
C2	0.1500 (4)	0.8244 (5)	0.4399 (4)	0.0853 (17)	
H2	0.1299	0.8878	0.4742	0.102*	
C3	0.2259 (4)	0.7777 (5)	0.5003 (5)	0.0903 (18)	
H3	0.2585	0.8102	0.5758	0.108*	
C4	0.2544 (4)	0.6830 (6)	0.4502 (5)	0.0860 (17)	
H4	0.3060	0.6512	0.4923	0.103*	
C5	0.2075 (3)	0.6351 (5)	0.3391 (4)	0.0723 (14)	
H5	0.2275	0.5708	0.3062	0.087*	
C6	0.1309 (3)	0.6806 (4)	0.2748 (4)	0.0573 (11)	
C7	0.0779 (3)	0.6306 (4)	0.1506 (4)	0.0614 (12)	
H7A	0.0201	0.6396	0.1453	0.074*	
H7B	0.0872	0.6762	0.0828	0.074*	
C8	0.0726 (2)	0.4235 (4)	0.1984 (3)	0.0492 (11)	
C9	0.0862 (2)	0.2966 (4)	0.1713 (3)	0.0477 (10)	
C10	0.0729 (3)	0.2155 (4)	0.2537 (4)	0.0645 (13)	
H10	0.0561	0.2411	0.3210	0.077*	
C11	0.0839 (4)	0.0971 (5)	0.2393 (5)	0.0802 (16)	
H11	0.0741	0.0441	0.2963	0.096*	
C12	0.1088 (4)	0.0581 (5)	0.1432 (5)	0.0833 (16)	
H12	0.1161	−0.0218	0.1338	0.100*	
C13	0.1236 (3)	0.1374 (4)	0.0576 (4)	0.0739 (14)	
H13	0.1417	0.1108	−0.0081	0.089*	
C14	0.1113 (2)	0.2551 (4)	0.0710 (3)	0.0506 (11)	
C15	0.1448 (3)	0.3013 (4)	−0.1168 (3)	0.0582 (12)	
H15A	0.1031	0.2491	−0.1691	0.070*	
H15B	0.1973	0.2602	−0.0900	0.070*	
C16	0.1516 (3)	0.4089 (4)	−0.1880 (4)	0.0583 (12)	
C17	0.1515 (3)	0.4860 (5)	−0.3908 (4)	0.0755 (15)	
H17A	0.1635	0.4531	−0.4634	0.091*	
H17B	0.1978	0.5355	−0.3464	0.091*	
C18	0.0744 (3)	0.5599 (4)	−0.4352 (4)	0.0633 (12)	
H18A	0.0655	0.5993	−0.3635	0.076*	
H18B	0.0820	0.6195	−0.4927	0.076*	
C19	0.1576 (7)	0.8011 (10)	0.8043 (10)	0.190 (4)	
H19A	0.1167	0.7735	0.7280	0.227*	
H19B	0.2120	0.7857	0.7965	0.227*	
C20	0.1476 (8)	0.9286 (12)	0.8154 (13)	0.220 (5)	
H20A	0.0967	0.9441	0.8332	0.330*	
H20B	0.1462	0.9660	0.7377	0.330*	
H20C	0.1932	0.9589	0.8825	0.330*	
N1	0.0936 (2)	0.5070 (3)	0.1313 (3)	0.0555 (9)	
H1A	0.1169	0.4881	0.0756	0.067*	
N2	0.1437 (2)	0.3914 (4)	−0.3083 (3)	0.0627 (10)	
H2A	0.1336	0.3217	−0.3385	0.075*	
N3	0.0000	0.4879 (5)	−0.5000	0.0538 (13)	
HN3	−0.004 (2)	0.437 (4)	−0.435 (4)	0.061 (12)*	
N4	0.0000	0.1418 (5)	0.5000	0.0692 (16)	
O1	0.04311 (19)	0.4475 (3)	0.2828 (3)	0.0665 (9)	
O2	0.1223 (2)	0.3375 (3)	−0.0115 (2)	0.0659 (8)	
O3	0.1642 (2)	0.5043 (4)	−0.1375 (3)	0.0879 (12)	
O4	0.1490 (5)	0.7383 (9)	0.9064 (8)	0.206 (3)	
H4A	0.1549	0.6662	0.8924	0.247*	
O5	−0.0303 (5)	0.2373 (6)	0.4705 (7)	0.086 (3)	0.50
O6	0.0685 (4)	0.1426 (7)	0.5858 (8)	0.100 (3)	0.50
O7	−0.0314 (6)	0.0489 (6)	0.4703 (11)	0.133 (4)	0.50

### Atomic displacement parameters (Å^2^)

**Table:**

	*U*^11^	*U*^22^	*U*^33^	*U*^12^	*U*^13^	*U*^23^
C1	0.106 (3)	0.055 (3)	0.059 (2)	0.008 (3)	0.028 (2)	0.008 (2)
C2	0.161 (5)	0.036 (2)	0.068 (3)	−0.008 (3)	0.049 (3)	−0.002 (2)
C3	0.118 (4)	0.083 (4)	0.061 (3)	−0.036 (3)	0.016 (3)	0.004 (3)
C4	0.084 (3)	0.094 (4)	0.073 (3)	−0.021 (3)	0.013 (3)	0.002 (3)
C5	0.083 (3)	0.073 (3)	0.062 (3)	−0.006 (3)	0.025 (2)	−0.003 (2)
C6	0.084 (3)	0.049 (2)	0.0445 (18)	0.001 (2)	0.0277 (19)	0.0087 (18)
C7	0.086 (3)	0.052 (3)	0.046 (2)	0.005 (2)	0.021 (2)	0.0030 (19)
C8	0.056 (2)	0.056 (3)	0.0357 (17)	0.001 (2)	0.0152 (16)	−0.0003 (18)
C9	0.057 (2)	0.053 (2)	0.0344 (16)	−0.0033 (19)	0.0158 (15)	0.0026 (17)
C10	0.085 (3)	0.066 (3)	0.049 (2)	0.008 (2)	0.030 (2)	0.013 (2)
C11	0.122 (4)	0.068 (3)	0.056 (2)	−0.004 (3)	0.035 (3)	0.013 (2)
C12	0.128 (4)	0.052 (3)	0.079 (3)	0.000 (3)	0.046 (3)	0.011 (2)
C13	0.108 (3)	0.063 (3)	0.065 (2)	0.002 (3)	0.048 (2)	0.003 (2)
C14	0.065 (2)	0.050 (2)	0.0426 (19)	−0.005 (2)	0.0251 (17)	0.0017 (18)
C15	0.082 (2)	0.063 (3)	0.0391 (17)	0.005 (2)	0.0330 (17)	−0.0049 (19)
C16	0.069 (2)	0.066 (3)	0.0455 (19)	−0.002 (2)	0.0255 (18)	0.004 (2)
C17	0.076 (2)	0.107 (4)	0.053 (2)	0.004 (3)	0.034 (2)	0.019 (3)
C18	0.090 (3)	0.055 (3)	0.053 (2)	−0.015 (2)	0.033 (2)	0.003 (2)
C19	0.183 (6)	0.205 (8)	0.190 (7)	−0.004 (7)	0.071 (6)	0.009 (7)
C20	0.218 (7)	0.219 (8)	0.221 (8)	−0.005 (7)	0.066 (6)	−0.043 (7)
N1	0.075 (2)	0.054 (2)	0.0423 (16)	0.0008 (18)	0.0244 (15)	0.0025 (16)
N2	0.088 (2)	0.067 (2)	0.0447 (16)	0.011 (2)	0.0376 (15)	0.0073 (17)
N3	0.078 (3)	0.051 (3)	0.038 (2)	0.000	0.027 (2)	0.000
N4	0.072 (3)	0.078 (4)	0.069 (3)	0.000	0.038 (3)	0.000
O1	0.0891 (18)	0.070 (2)	0.0545 (14)	−0.0011 (17)	0.0420 (14)	−0.0032 (15)
O2	0.113 (2)	0.0502 (18)	0.0509 (13)	0.0028 (16)	0.0492 (14)	0.0024 (13)
O3	0.125 (3)	0.080 (2)	0.0584 (17)	−0.033 (2)	0.0280 (18)	−0.0035 (18)
O4	0.236 (6)	0.214 (7)	0.200 (5)	−0.015 (5)	0.117 (5)	0.006 (5)
O5	0.127 (7)	0.065 (4)	0.079 (5)	0.017 (4)	0.049 (5)	0.018 (4)
O6	0.113 (5)	0.063 (5)	0.138 (7)	0.013 (5)	0.061 (5)	−0.002 (5)
O7	0.185 (11)	0.070 (5)	0.165 (8)	−0.055 (5)	0.084 (7)	−0.062 (6)

### Geometric parameters (Å, °)

**Table:**

C1—C6	1.367 (7)	C16—O3	1.214 (6)
C1—C2	1.402 (7)	C16—N2	1.322 (5)
C1—H1	0.9300	C17—N2	1.452 (6)
C2—C3	1.365 (8)	C17—C18	1.506 (7)
C2—H2	0.9300	C17—H17A	0.9700
C3—C4	1.371 (8)	C17—H17B	0.9700
C3—H3	0.9300	C18—N3	1.493 (5)
C4—C5	1.364 (7)	C18—H18A	0.9700
C4—H4	0.9300	C18—H18B	0.9700
C5—C6	1.378 (6)	C19—O4	1.392 (11)
C5—H5	0.9300	C19—C20	1.475 (14)
C6—C7	1.512 (6)	C19—H19A	0.9700
C7—N1	1.464 (6)	C19—H19B	0.9700
C7—H7A	0.9700	C20—H20A	0.9600
C7—H7B	0.9700	C20—H20B	0.9600
C8—O1	1.226 (5)	C20—H20C	0.9600
C8—N1	1.327 (5)	N1—H1A	0.8600
C8—C9	1.511 (6)	N2—H2A	0.8600
C9—C10	1.372 (6)	N3—C18^i^	1.493 (5)
C9—C14	1.398 (5)	N3—HN3	0.95 (4)
C10—C11	1.380 (8)	N4—O7^ii^	1.186 (8)
C10—H10	0.9300	N4—O7	1.186 (8)
C11—C12	1.344 (8)	N4—O5^ii^	1.205 (8)
C11—H11	0.9300	N4—O5	1.205 (8)
C12—C13	1.395 (7)	N4—O6	1.258 (7)
C12—H12	0.9300	N4—O6^ii^	1.258 (7)
C13—C14	1.374 (7)	O4—H4A	0.8491
C13—H13	0.9300	O5—O5^ii^	1.035 (14)
C14—O2	1.368 (5)	O5—O6^ii^	1.316 (10)
C15—O2	1.406 (5)	O6—O7^ii^	1.298 (12)
C15—C16	1.486 (6)	O6—O5^ii^	1.316 (10)
C15—H15A	0.9700	O7—O7^ii^	1.066 (18)
C15—H15B	0.9700	O7—O6^ii^	1.298 (12)
			
C6—C1—C2	121.2 (5)	O2—C15—H15B	110.3
C6—C1—H1	119.4	C16—C15—H15B	110.3
C2—C1—H1	119.4	H15A—C15—H15B	108.6
C3—C2—C1	118.7 (5)	O3—C16—N2	123.7 (4)
C3—C2—H2	120.7	O3—C16—C15	121.6 (4)
C1—C2—H2	120.7	N2—C16—C15	114.8 (4)
C2—C3—C4	120.3 (5)	N2—C17—C18	112.6 (4)
C2—C3—H3	119.8	N2—C17—H17A	109.1
C4—C3—H3	119.8	C18—C17—H17A	109.1
C5—C4—C3	120.4 (5)	N2—C17—H17B	109.1
C5—C4—H4	119.8	C18—C17—H17B	109.1
C3—C4—H4	119.8	H17A—C17—H17B	107.8
C4—C5—C6	120.9 (5)	N3—C18—C17	111.7 (4)
C4—C5—H5	119.5	N3—C18—H18A	109.3
C6—C5—H5	119.5	C17—C18—H18A	109.3
C1—C6—C5	118.5 (4)	N3—C18—H18B	109.3
C1—C6—C7	119.3 (4)	C17—C18—H18B	109.3
C5—C6—C7	122.2 (4)	H18A—C18—H18B	107.9
N1—C7—C6	114.5 (4)	O4—C19—C20	113.2 (12)
N1—C7—H7A	108.6	O4—C19—H19A	108.9
C6—C7—H7A	108.6	C20—C19—H19A	108.9
N1—C7—H7B	108.6	O4—C19—H19B	108.9
C6—C7—H7B	108.6	C20—C19—H19B	108.9
H7A—C7—H7B	107.6	H19A—C19—H19B	107.7
O1—C8—N1	121.3 (4)	C19—C20—H20A	109.5
O1—C8—C9	119.5 (4)	C19—C20—H20B	109.5
N1—C8—C9	119.2 (3)	H20A—C20—H20B	109.5
C10—C9—C14	117.6 (4)	C19—C20—H20C	109.5
C10—C9—C8	116.4 (4)	H20A—C20—H20C	109.5
C14—C9—C8	126.0 (4)	H20B—C20—H20C	109.5
C9—C10—C11	121.7 (5)	C8—N1—C7	121.0 (4)
C9—C10—H10	119.1	C8—N1—H1A	119.5
C11—C10—H10	119.1	C7—N1—H1A	119.5
C12—C11—C10	120.3 (5)	C16—N2—C17	122.1 (4)
C12—C11—H11	119.9	C16—N2—H2A	118.9
C10—C11—H11	119.9	C17—N2—H2A	118.9
C11—C12—C13	120.0 (5)	C18—N3—C18^i^	113.2 (5)
C11—C12—H12	120.0	C18—N3—HN3	102 (2)
C13—C12—H12	120.0	C18^i^—N3—HN3	117 (2)
C14—C13—C12	119.6 (5)	O7^ii^—N4—O5^ii^	127.9 (6)
C14—C13—H13	120.2	O7—N4—O5	127.9 (6)
C12—C13—H13	120.2	O7—N4—O6	116.6 (8)
O2—C14—C13	122.8 (4)	O5—N4—O6	114.7 (7)
O2—C14—C9	116.4 (4)	O7^ii^—N4—O6^ii^	116.6 (8)
C13—C14—C9	120.8 (4)	O5^ii^—N4—O6^ii^	114.7 (7)
O2—C15—C16	106.9 (4)	C14—O2—C15	119.3 (3)
O2—C15—H15A	110.3	C19—O4—H4A	107.3
C16—C15—H15A	110.3		
			
C6—C1—C2—C3	1.2 (8)	C12—C13—C14—O2	−178.0 (4)
C1—C2—C3—C4	−1.2 (8)	C12—C13—C14—C9	1.7 (7)
C2—C3—C4—C5	0.5 (9)	C10—C9—C14—O2	178.5 (4)
C3—C4—C5—C6	0.1 (8)	C8—C9—C14—O2	−1.8 (5)
C2—C1—C6—C5	−0.5 (7)	C10—C9—C14—C13	−1.2 (6)
C2—C1—C6—C7	−179.8 (5)	C8—C9—C14—C13	178.5 (4)
C4—C5—C6—C1	−0.1 (7)	O2—C15—C16—O3	23.1 (6)
C4—C5—C6—C7	179.2 (5)	O2—C15—C16—N2	−157.5 (4)
C1—C6—C7—N1	−155.0 (4)	N2—C17—C18—N3	−56.5 (5)
C5—C6—C7—N1	25.7 (7)	O1—C8—N1—C7	−3.7 (5)
O1—C8—C9—C10	−7.0 (5)	C9—C8—N1—C7	177.2 (3)
N1—C8—C9—C10	172.1 (4)	C6—C7—N1—C8	69.5 (5)
O1—C8—C9—C14	173.3 (3)	O3—C16—N2—C17	1.6 (7)
N1—C8—C9—C14	−7.6 (5)	C15—C16—N2—C17	−177.8 (4)
C14—C9—C10—C11	0.2 (6)	C18—C17—N2—C16	−78.9 (5)
C8—C9—C10—C11	−179.6 (4)	C17—C18—N3—C18^i^	−169.9 (4)
C9—C10—C11—C12	0.4 (8)	C13—C14—O2—C15	1.8 (6)
C10—C11—C12—C13	0.0 (9)	C9—C14—O2—C15	−177.9 (3)
C11—C12—C13—C14	−1.1 (8)	C16—C15—O2—C14	179.8 (3)

Symmetry codes: (i) −*x*, *y*, −*z*−1; (ii) −*x*, *y*, −*z*+1.

### Hydrogen-bond geometry (Å, °)

**Table:**

*D*—H···*A*	*D*—H	H···*A*	*D*···*A*	*D*—H···*A*
N3—HN3···O5^iii^	0.95 (4)	2.49 (5)	2.905 (9)	106 (3)
N3—HN3···O5^iv^	0.95 (4)	2.41 (5)	2.905 (9)	112 (3)
N3—HN3···O1^iv^	0.95 (4)	2.01 (4)	2.780 (3)	137 (4)
O4—H4A···O3^v^	0.85	1.89	2.740 (11)	178
N1—H1A···O2	0.86	1.99	2.645 (5)	132
N2—H2A···O5^iv^	0.86	2.14	2.813 (9)	135
N2—H2A···O6^iii^	0.86	2.35	3.185 (9)	163

Symmetry codes: (iii) *x*, *y*, *z*−1; (iv) −*x*, *y*, −*z*; (v) *x*, *y*, *z*+1.
